# VSIG2 promotes malignant progression of pancreatic ductal adenocarcinoma by enhancing LAMTOR2-mediated mTOR activation

**DOI:** 10.1186/s12964-023-01209-x

**Published:** 2023-08-25

**Authors:** Jichuan Xu, Gang Quan, Wei Huang, Jianxin Jiang

**Affiliations:** 1 Department of Hepatobiliary, Pancreatic and Splenic Surgery, Affiliated Dongguan Hospital, Southern Medical University (Dongguan People’s Hospital), 78 Wandao Road, Wanjiang Street, Dongguan City, Guangdong Province 523058 People’s Republic of China; 2 Department of Hepatobiliary Surgery, Renmin Hospital of Wuhan University, Wuhan, Hubei China

**Keywords:** Pancreatic ductal adenocarcinoma, Malignant progression, V-set and immunoglobulin domain containing 2 (VSIG2), Late endosomal/lysosomal adaptor, MAPK and MTOR activator 2 (LAMTOR2), Mechanistic target of rapamycin (mTOR)

## Abstract

**Background:**

Pancreatic ductal adenocarcinoma (PDAC) is one of the most intractable malignancies to overcome clinically due to its insidious onset as well as rapid progression. It is urgent to seek new diagnostic markers and therapeutic targets in order to furthest ameliorate the prognosis of patients with PDAC. V-set and immunoglobulin domain containing 2 (VSIG2) belongs to immunoglobulin superfamily (IgSF), which function as coinhibitory molecule to mediate immune evasion of tumors. Nevertheless, the role of VSIG2 in PDAC and related mechanism still keep unclear.

**Methods:**

Different expression of VSIG2 in PDAC tissues and cells were detected by bioinformatic analysis, immunohistochemistry, real-time quantitative PCR as well as western blotting. CCK-8, colony formation, Transwell assay, and scratch experiment were utilized to assess proliferation, invasion and migration properties of PDAC cells. The relationship of VSIG2 with late endosomal/lysosomal adaptor, MAPK and MTOR activator 2 (LAMTOR2) and mechanistic target of rapamycin (mTOR) was identified using mass spectrometry, co-immunoprecipitation and immunofluorescence. GO and KEGG enrichment analysis were performed for further pathway verification using western blotting. Additionally, subcutaneous xenograft tumor model and clinical samples analysis were implemented to further elucidate the oncogenic effect of VSIG2 on PDAC in vivo and clinically.

**Results:**

VSIG2 was highly expressed in PDAC tissues and cells. Overexpression of VSIG2 facilitated the proliferation, invasion and migration abilities of PDAC cells, while VSIG2-inhibition exerted opposite effects. Mechanistically, VSIG2 could simultaneously bind to LAMTOR2 and mTOR, thereby enhancing interaction between two molecules, which resulted in elevated phosphorylation-modificatory activation of mTOR and downstream key molecules. Clinically, up-regulation of VSIG2 was positively associated with advanced stage, overall survival and disease-free survival of PDAC patients.

**Conclusions:**

Our study disclosed that VSIG2 was overexpressed in PDAC, which promoted the proliferation, invasion and metastasis. Mechanically, VSIG2 acted as a scaffold to recruit LAMTOR2 and mTOR simultaneously, stabilize the interaction between them, thus enhancing LAMTOR2-mediated mTOR phosphorylated activation. Collectively, VSIG2 could be exploited as a biomarker for diagnosis and prognosis monitor of PDAC in the future, meanwhile, targeting VSIG2 in PDAC management is expected to be a novel strategy. Video Abstract.

Video Abstract

**Supplementary Information:**

The online version contains supplementary material available at 10.1186/s12964-023-01209-x.

## Introduction

As a highly malignant solid neoplasm of the digestive system, the morbidity and mortality of pancreatic ductal adenocarcinoma (PDAC) are increasing by years worldwide, which has become the fourth leading cause of cancer-related deaths [1]. Despite the continuous sophistication of detection technology and treatment methods, the five-year survival rate of patients with PDAC is still lower than 10%, while the median survival time is only 3-6 months [2, 3]. PDAC is poorly differentiated in cell level, which acquires the capacity of local invasion and distant metastasis attributed to tolerance to hypoxia microenvironment [3]. It is untoward to make diagnosis because of silent clinical manifestations in early pathological period, while 80-85% of patients are confirmed in the advanced stage of lacking surgical indications when hospitalized owe to abdominal or mid-back pain, jaundice, cachexia as well as other manifestations [3, 4]. Meanwhile, PDAC is characterized by chemoresistance, surgical resection is still the only possible cure [3, 5]. Therefore, in order to enrich effective treatment methods, thereby improving the prognosis, we need to explore the biological mechanism of the occurrence and development of PDAC and seek potential molecular targets.

Mechanistic target of rapamycin (mTOR), as a serine/threonine kinase, is the dominating component of mTOR complex (mTORC1/2), which could phosphorylate ribosomal protein S6 kinase (S6K), eukaryotic translation initiation factor 4E binding protein 1 (4EBP1) and other target proteins to modulate protein synthesis, nutrient metabolism, cell growth and migration [6]. Late endosomal/lysosomal adaptor, MAPK and MTOR activator 2 (LAMTOR2) is part of the Ragulator complex which involved in amino acid sensing and activation of mTORC1 in combination with Rag GTPases [7, 8]. The activated Ragulator-Rag complex containing LAMTOR2 acts as a scaffold to recruit and activate mTORC1 [9]. Activation of the mTOR signaling pathway can accelerate neoplasm growth and metastasis [6]. Several mTOR inhibitors, including rapamycin and its derivatives, ATP competitive inhibitors, and dual PI3K/mTOR inhibitors, have been reported for the treatment of advanced renal cell carcinoma, pancreatic neuroendocrine tumor, breast cancer, gastric cancer, hepatocellular carcinoma, non-small cell lung cancer, endometrial carcinoma, and mantle cell lymphoma [10–13].

V-set and immunoglobulin domain containing 2 (VSIG2) gene is located in the long chain region 2 of chromosome 11 [14]. Its encoded protein belongs to the B7 family protein, which is also a member of the immunoglobulin superfamily (IgSF) [14, 15]. VSIG2 can perform a function as a novel immune checkpoint to regulate immune response and act as a cooperative ligand to inhibit T cell function [14, 16]. In addition, VSIG2 protein is involved in B cell and M1 macrophage infiltration [17]. In recent years, various researches have demonstrated that VSIG2 expression is positively correlated with the progression of multiple diseases, such as FECD, IBS-D, acute tubular injury and interstitial fibrosis/tubular atrophy, and incident heart failure [18–21]. It is worth noting that VSIG2 expression is positively associated with the development and poor prognosis of AML, primary lung adenocarcinoma, and PDAC, while VSIG2 suppresses muscle invasive bladder cancer as well as COAD [17, 22–25]. Our study elucidated another mechanism of VSIG2 in promoting the malignant progression of PDAC besides mediating the tumor immune response, and provided an experimental basis for further evaluation of its value as a molecular marker and therapeutic target.

## Materials and methods

### Cell culture

Human pancreatic cancer cell lines PANC-1, MIA PaCa-2, SW 1990, AsPC-1, BxPC-3 and normal human pancreatic ductal epithelial (HPDE) cell line were all purchased from American Type Culture Collection (ATCC, USA). Above all, PANC-1, MIA PaCa-2, and SW 1990 cell lines were cultured by Dulbecco’s Modified Eagle Medium (DMEM) high glucose medium (Hyclone, USA) with 10% fetal bovine serum (FBS) (Gibco, USA), while HPDE, AsPC-1, BxPC-3 cell lines were sustained by RPMI 1640 medium (Hyclone, USA) with 10% FBS. And all the cell lines were cultured in cell incubator at 37° C and 5% CO_2_.

### RNA isolation and real-time quantitative PCR (RT-qPCR)

RNA isolation was performed by TRIzol method. The ratio of OD_260_/OD_280_ detected by spectrophotometer was between 1.8-2.0, and the RNA extract could be used for subsequent reverse transcription PCR, while RNA concentration was recorded at the same time. The cDNA synthesis reaction was carried out according to the reagent instructions. The reaction system consisted of 5μl 2X Universal SYBR Green Fast qPCR Mix (ABclonal, China), 0.2μl Forward Primer, 0.2μl Reverse Primer (Sango Biotech, China), 0.5μl cDNA and 4.1μl DEPC water. PCR reaction conditions were set as follows: pre-denaturation 95° C, 30s; denaturation 95° C, 10s; annealing extension 60° C, 30s and 40 cycles in total. The internal control was β-actin. The 2^−ΔΔCt^ method was utilized to evaluate the relative expression. Supplementary table 1 lists the details of all PCR primer sequences in this study. The experiment was repeated three times.

### Protein extraction and Western blot

Protein extract solution split cells after centrifugation which were then broken by ultrasound for 6 times, 5s each time. After centrifugation with 12000r/min at 4° C for 15min, part of the supernatant was taken for protein quantification, and the rest was added with 5×loading buffer occupying a quarter of the volume of the supernatant while boiled in a metal bath at 95° C for 10min. BCA method was utilized to detect the protein concentration. SDS-PAGE gel electrophoresis was performed at 80V for 2h, followed by transferring onto PVDF membrane at constant 300mA for 2h. Above PVDF membrane was blocked for 25min, followed by incubation with primary antibody at 4° C for 14-16h. The membrane was washed by TBST for three times, while subsequently incubated with secondary antibody at room temperature for 2h. The membrane was washed by TBST again for three times. Ultimately, ECL chemiluminescence method was employed for exposure and gray analysis. The experiment was repeated three times. Antibodies used in this study are listed in Supplementary Table 2.

### Transfection

3μg plasmid and 10μl P3000^TM^ Reagent (ThermoFisher, USA) were added to 250μl OPTI-MEM medium (Gibco, USA) and incubated for 10min at room temperature. Simultaneously, 5μl Lipofectamine 3000 (ThermoFisher, USA) was added to 250μl OPTI-MEM medium and incubated for 10min at room temperature. The above two mixtures were mixed and added to the conventional medium in the six-well plate, followed by change of medium after 24h.

### Cell counting kit-8 (CCK-8)

The cells in logarithmic growth stage were selected and seeded in a 96-well plate at a rate of 1×10^3^ cells/well, and each sample was set up with 3 subsidiary wells while cultured in the incubator. The CCK-8 solution (Dojindo, Japan) was diluted according to the ratio of medium : CCK-8 = 1 : 10, and then 110μl of above mixed medium was added to each well to replace conventional medium, meanwhile, the 96-well plate got further incubation for 1-4h. After adjusting the incubation time, OD_450_ value was detected by microplate reader while drawing the cell viability curve. The experiment was repeated three times.

### Colony formation

Five hundred cells were inoculated in six-well plate, followed by timely replacement of the medium according to the cell morphology and growth condition. The above six-well plate was cultivated for 10-14 days until the cell mass appeared at the bottom of the plate. After washing by PBS twice, 4% paraformaldehyde was added to fix for 15min. 1% crystal violet was subsequently added in the plate to stain for further 30min while the number of colony formation was counted and recorded. The experiment was repeated three times.

### Transwell

Complete medium was added to the lower chamber of the 24-well plate (Corning, USA), and serum-free medium was added to the upper chamber (Corning, USA). 4-5×10^4^ cells were inoculated in each chamber, while the 24-well plate was shaken and cultured in the incubator for 24-36h. 4% paraformaldehyde was added to the lower chamber to fix for 20min. After cleaning again with PBS, 1% crystal violet was taken and stained for 30min. Under the microscope, 3-6 fields were randomly selected for observation and counting. The experiment was repeated three times. When the invasion assay was carried out, BD Matrigel glue (Becton Dickinson, USA) should be laid in advance. Other experimental procedures were the same as the migration assay.

### Scratch assay

Cells at logarithmic growth stage were inoculated in a six-well plate. When the cell density reached 90%, cells were paddled perpendicularly at a constant speed with a 200μl gun head, cleaned twice with PBS, photographed under the microscope, and the scratch area of 0h was recorded. After the serum-free medium was added for another 24h, the scratch area was photographed again to record. The cell mobility was calculated by comparing the change of scratch area. The experiment was repeated three times.

### Co‐immunoprecipitation (Co‐IP)

The cell lysates were incubated with the primary antibody at 4° C overnight. Subsequently, the protein A/G Sepharose beads (Santa Cruz, USA) were added to the above mixture. Then, the interaction proteins were recognized by immunoblotting via incubation with specific antibodies.

### Immunofluorescence

The coverslip was fixed with 4% paraformaldehyde for 15 min and soaked with PBS three times for 3min each time. The cells were permeated by 0.5% Triton X-100 at room temperature for 15min and then soaked again by PBS. 5% normal serum was added to the coverslip to block at room temperature for 1h, followed by incubation with primary antibody at 4° C overnight. Fluorescent secondary antibody was added to incubate at 37° C for 1h away from light. DAPI was subsequently added to incubate for 5min away from light. Finally, the coverslip was sealed with liquid seal, while the images were observed and collected under fluorescence microscope.

### Immunohistochemistry (IHC)

The paraffin sections were dewaxed to water and incubated with 3% H_2_O_2_ for 8min at room temperature, then rinsed with distilled water and soaked twice in PBS for 5min each time. The above sections were blocked with 10% normal goat serum diluted in PBS and incubated at room temperature for 10min. After the serum was discarded, the working solution of primary antibody was added and the samples were kept at 4° C overnight. Subsequently, appropriate amounts of biotin-labeled secondary antibody working solution and horseradish enzyme working solution were added successively and incubated at 37° C for 20min. Before and after the addition of working solution, the sections were washed with PBS for three times, 5min each time, and the color was developed with DAB for 15min. Finally, the sections were thoroughly rinsed with double steaming water, counterstained, dehydrated, transparent and sealed successively, while followed by visual analysis of the samples.

### Subcutaneous xenograft tumor model

Female BALB/c nude mice were selected for subcutaneous tumor transplantation model experiment. 100 μL of 1×10^6^ PDAC cells were injected subcutaneously into the right axilla of BALB/c nude mice. Tumor diameter was recorded once every three days from the beginning of subcutaneous transplantation and tumor mass was recorded after tumor dissection.

### Bioinformatics

TIMER2.0 (http://timer.comp-genomics.org/) database was used for pan-cancer analysis of VSIG2. GEPIA (http://gepia.cancer-pku.cn/) and GEO (https://www.ncbi.nlm.nih.gov/) databases predicted the expression of VSIG2 in normal pancreas and pancreatic cancer. TCGA (https://portal.gdc.cancer.gov/) and GEO (https://www.ncbi.nlm.nih.gov/) databases were performed for paired sample analysis of VSIG2 expression levels in pancreatic cancer and para-cancer tissues. AlphaFold Protein Structure Database (https://alphafold.ebi.ac.uk/) predicted the three-dimensional structure of proteins. KEGG and GO enrichment analysis were performed on the protein spectrum results. Kaplan-Meier (https://kmplot.com/analysis/) database predicted the survival prognosis of VSIG2 on pancreatic cancer patients.

### The collection of pancreatic cancer and adjacent tissue samples

Clinical data from 62 pancreatic cancer patients were tracked in Renmin Hospital of Wuhan University (Wuhan, Hubei, China). All experiments about pancreatic cancer tissue samples were approved by the Ethics Committee of Renmin Hospital of Wuhan University (Wuhan, Hubei, China).

### Statistical analysis

GraphPad Prism 9 software was utilized to analyze the data, and the statistical results of samples were expressed as mean ± standard deviation. Student’s *t* test was used for comparison of two independent samples, while Fisher test (one-way ANOVA) was adopted for comparison of multiple samples. In addition, SNK-q test was taken for multiple comparisons among multiple samples. The discrepancy between Kaplan-Meier curves was analyzed by log-rank test. *P*<0.05 was considered statistically significant.

## Results

### VSIG2 was highly expressed in PDAC tissues and cells

**Fig. 1:**
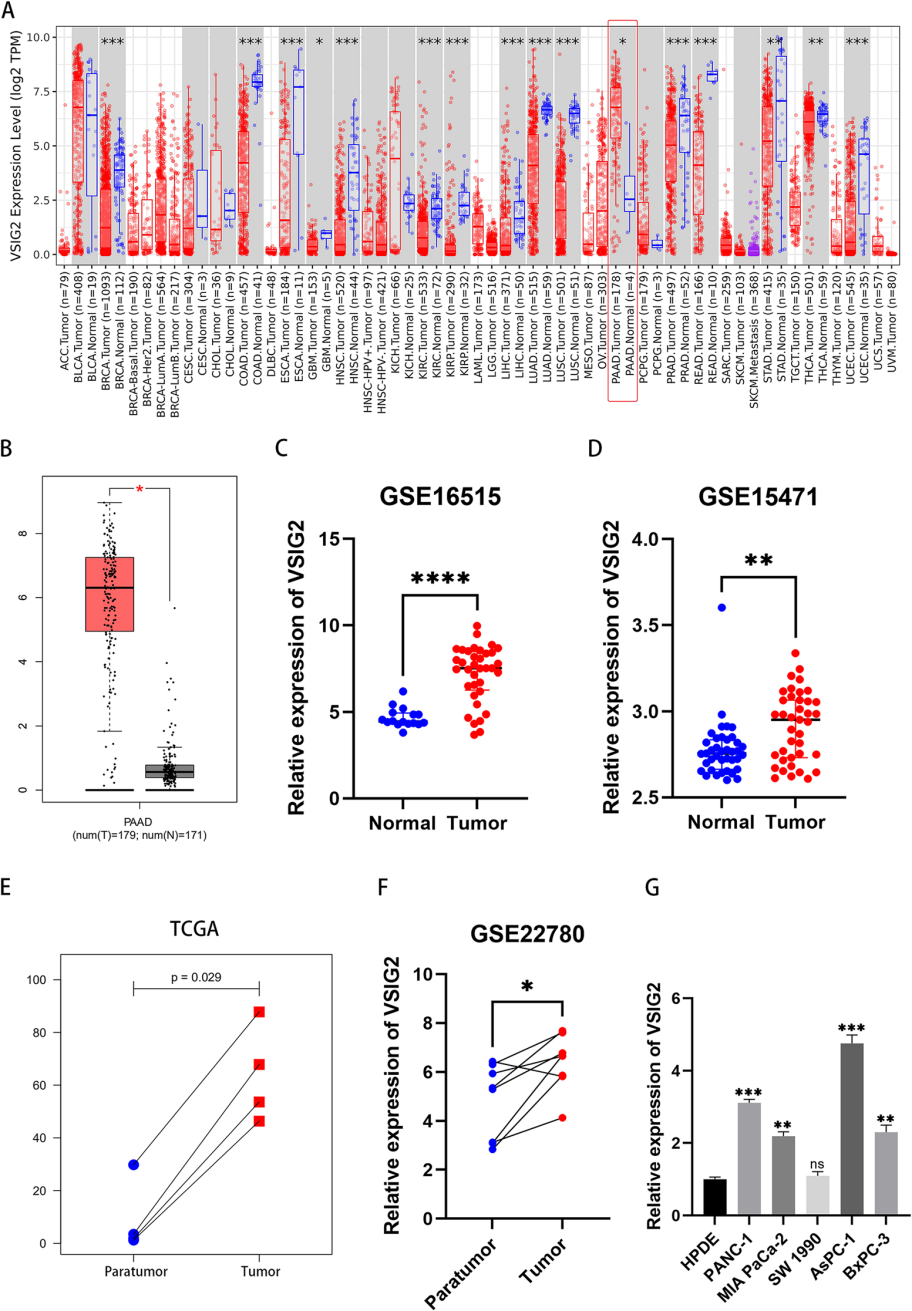
VSIG2 was overexpressed in PDAC tissues and cells. **A** Pan-cancer analysis of VSIG2 in multiple malignancies through TIMER2.0 (http://timer.comp-genomics.org/) database. **B**, **C**, **D** Differential expression analysis of VSIG2 in PDAC and normal pancreatic tissues through GEPIA (http://gepia.cancer-pku.cn/) and GEO (https://www.ncbi.nlm.nih.gov/) databases. **E**, **F** Paired sample analysis of VSIG2 expression levels in PDAC and para-cancer tissues through TCGA (https://portal.gdc.cancer.gov/) and GEO (https://www.ncbi.nlm.nih.gov/) databases. **G** Detection of VSIG2 relative expression in human pancreatic ductal epithelial and five PDAC cell lines by RT-qPCR

TIMER2.0 database was used for pan-cancer analysis, which showed significant differential expression of VSIG2 in BRCA, ESCA, HNSC, KIRC, KIRP, LUAD, LUSC, PAAD, STAD, UCEC, THCA, READ, PRAD, LIHC, GBM, and COAD (Fig. 1A). Ulteriorly, we found that VSIG2 was uniquely overexpressed in PDAC in combination with the analysis of GEPIA database (Fig. 1B). Besides, it was identified that VSIG2 was up-regulated in PDAC through analyzing GSE16515 and GSE15471 datasets (Fig. 1C, D). Similarly, VSIG2 was significantly overexpressed in tumor compared to para-tumor according to TCGA and GSE22780 dataset (Fig. 1E, F). The results of RT-qPCR and western blot uncovered that VSIG2 distinct elevation in PANC-1 and AsPC-1 cells, which were designated for subsequent experiments (Fig. 1G, Fig. S1A).

### VSIG2 accelerated the malignant biological behaviors of PDAC cells

**Fig. 2:**
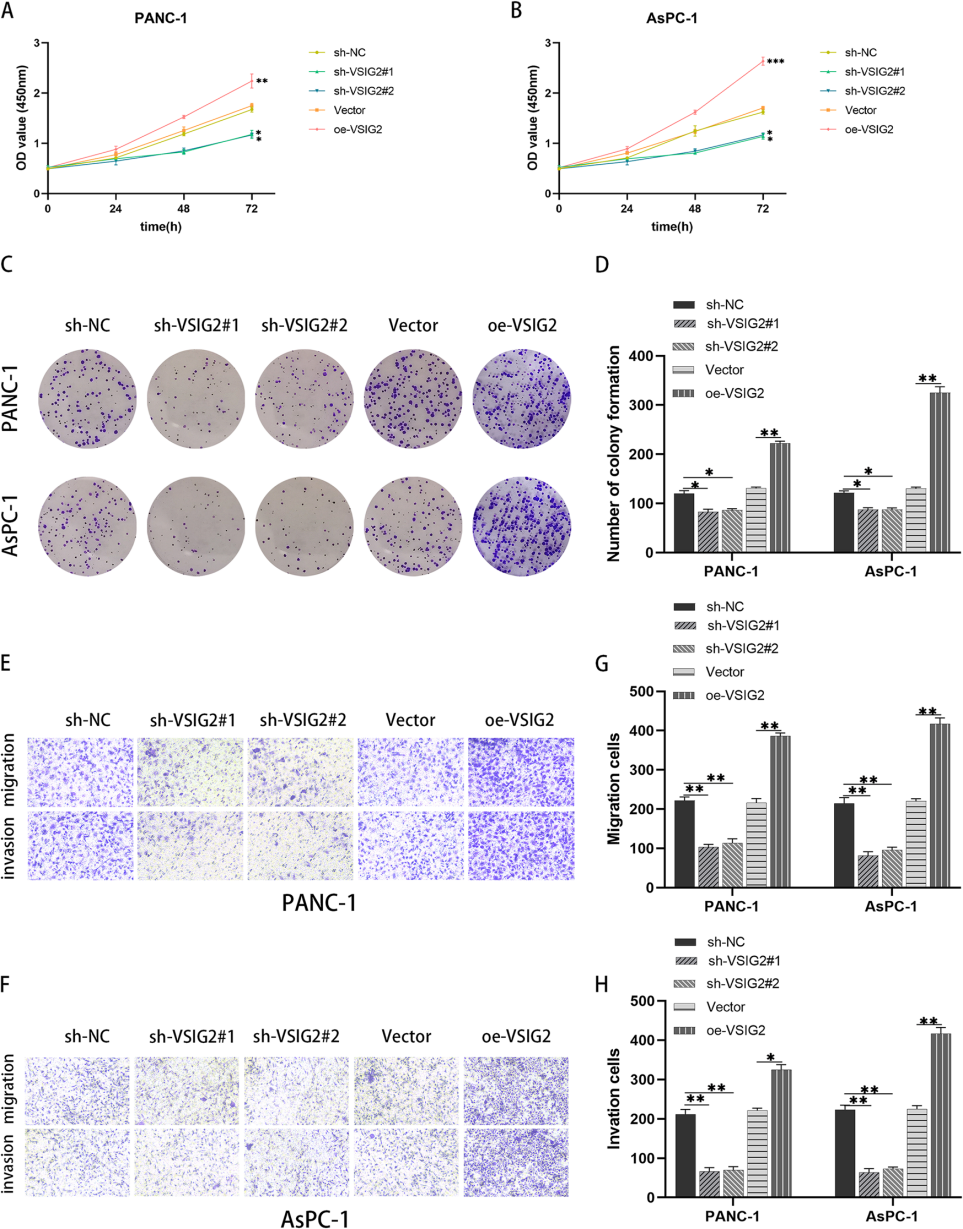
Effects of VSIG2 on PDAC cell proliferation, invasion and migration. **A**, **B** The proliferation ability of PANC-1 and AsPC-1 cells was detected by CCK-8 assay, and the comparison between experimental and control groups was adopted by OD_450_ value at 72h, *P<0.05, **P<0.01, ***P<0.001. **C**, **D** The proliferation ability of VSIG2 knockdown and overexpression groups in PANC-1 and AsPC-1 cells was detected by colony formation assay, *P<0.05, **P<0.01. **E**, **F**, **G**, **H** The invasion and migration properties of experimental and control groups in PANC-1 and AsPC-1 cells were clarified by Transwell assay, *P<0.05, **P<0.01

**Fig. 3:**
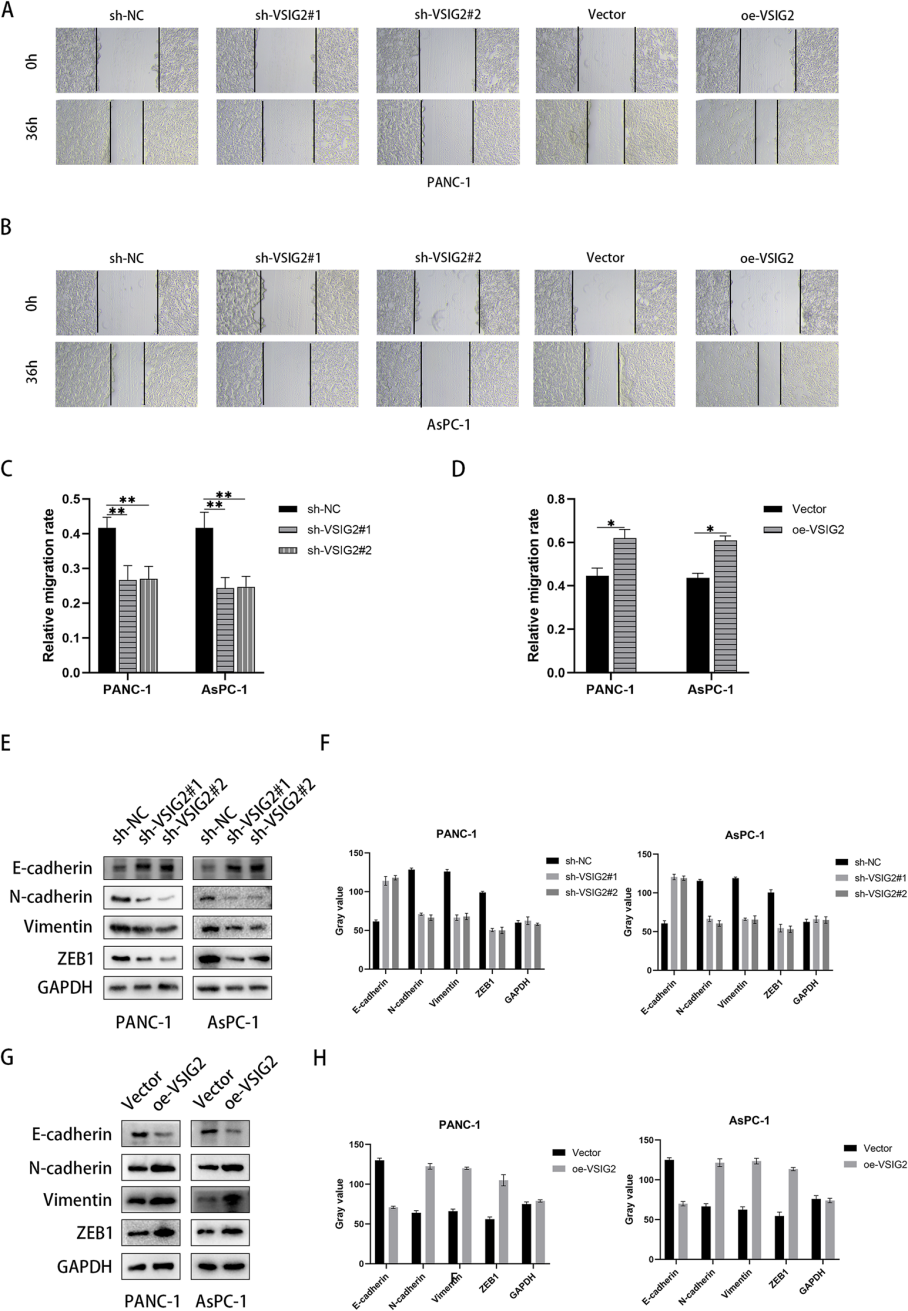
The role of VSIG2 in cell migration and epithelial-mesenchymal transition (EMT) of PDAC. **A**, **B**, **C**, **D** The migration ability of PANC-1 and AsPC-1 cells with VSIG2 down-regulation and overexpression was identified by evaluating relative migration rate via scratch assay, *P<0.05, **P<0.01. **E**, **F**, **G**, **H** The levels of EMT after knocking down and up-regulating VSIG2 expression in PANC-1 and AsPC-1 cells were verified by immunoblotting through assessing proteins expression of EMT related indicators including E-cadherin, N-cadherin, Vimentin, and ZEB1, when GAPDH was used as a loading control

After the transfection efficiency of VSIG2 was verified by immunoblotting (Fig. S1B, C), the results of CCK-8 showed that VSIG2 depletion impaired the proliferation of PDAC cells, whereas up-regulated VSIG2 led to the opposite phenotype (Fig. 2A, B). Similarly, colony formation assay was performed to further examine cell proliferation, which showed that VSIG2 inhibition induced less colonies regarding to PDAC cells, indicating the proliferation was hampered. On the contrary, over-expressed VSIG2 displayed more colonies, which promoted the proliferation of PDAC cells (Fig. 2C, D). Additionally, Transwell assay and scratch assay were performed to detect the abilities of migration and invasion about PDAC cells, which revealed that VSIG2 depletion reduced the migration and invasion, instead that the migration and invasion were enhanced after VSIG2 overexpression (Fig. 2E-H and Fig. 3A-D). The epithelial indicator E-cadherin was overexpressed in VSIG2 knock-down PDAC cells, while the mesenchymal indicators including N-cadherin, Vimentin and ZEB1 were decreased. Nevertheless, the expression of epithelial and mesenchymal transition (EMT) indicators displayed opposite phenomenon in up-regulated VSIG2 cells (Fig. 3E-H).

### VSIG2 interacted with LAMTOR2 and mTOR

**Fig. 4:**
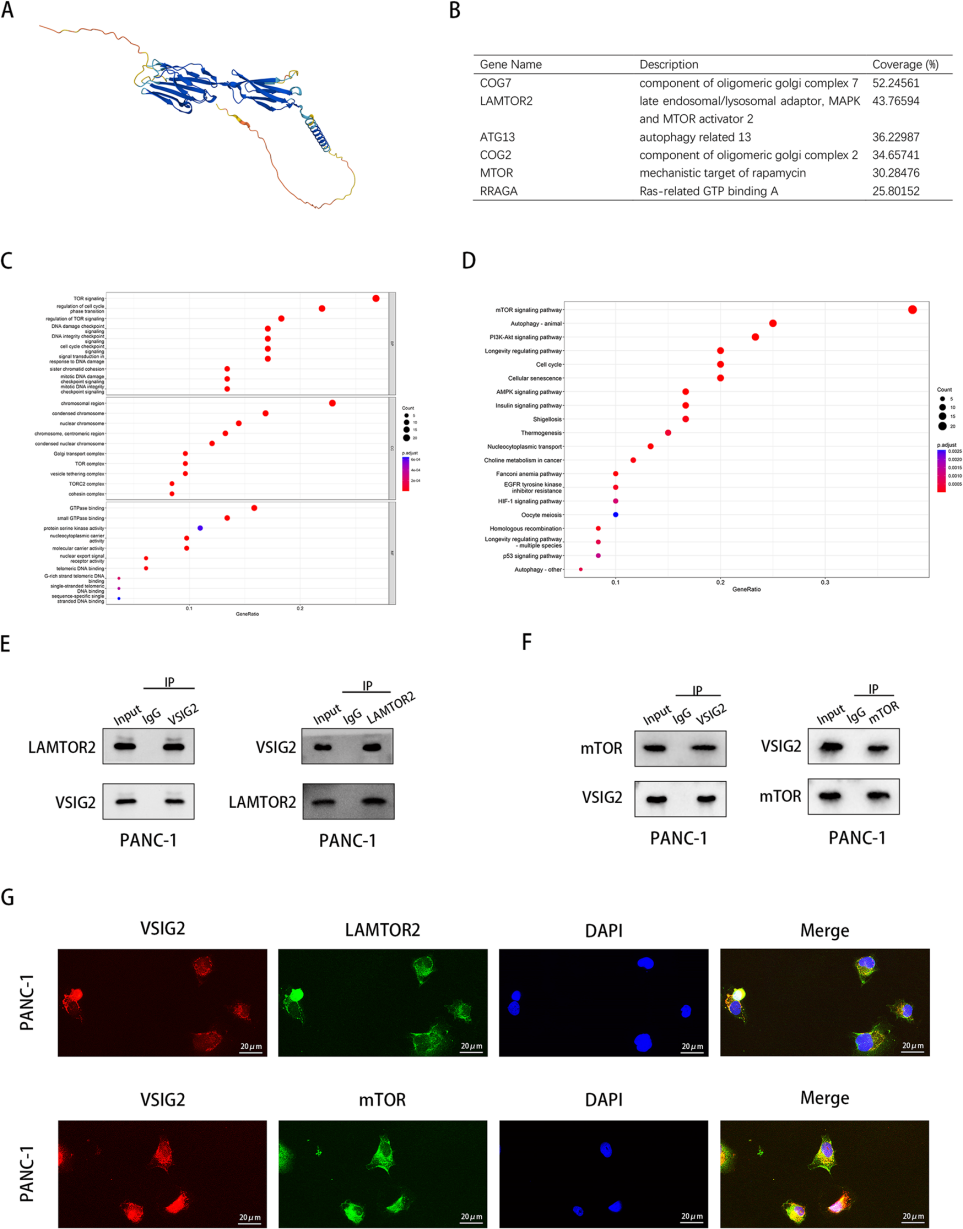
VSIG2 interacted with LAMTOR2 and mTOR. **A** AlphaFold Protein Structure Database (https://alphafold.ebi.ac.uk/) predicted the three-dimensional structure of VSIG2. **B** The results of VSIG2 mass spectrometry showed the possible interacting proteins, including LAMTOR2 and mTOR. **C** Proteins in VSIG2 mass spectrometry were analyzed from biological program, cellular condition and molecular function aspects by Gene Ontology (GO). **D** Kyoto Encyclopedia of Genes and Genomes (KEGG) predicted the related signaling pathway according to the results of VSIG2 mass spectrometry. **E** The interaction between VSIG2 and LAMTOR2 was testified by endogenic co-immunoprecipitation (Co-IP) in PANC-1 cells. **F** The interaction between VSIG2 and mTOR was verified by endogenic Co-IP assay in PANC-1 cells. **G** The interaction of VSIG2 with LAMTOR2 and mTOR and its spatial localization in PANC-1 cells were observed by immunofluorescence

We predicted the three-dimensional structure of VSIG2 protein via AlphaFold database (Fig. 4A). The results of VSIG2 protein spectrum showed that the top six proteins in the abundance ratio were COG7, LAMTOR2, ATG13, COG2, MTOR, RRAGA (Fig. 4B). Subsequently, GO enrichment analysis about VSIG2 mass spectrum showed that VSIG2-related proteins were involved in TOR signaling biological program with GTPase binding molecular function in all probability (Fig. 4C). Also, KEGG enrichment analysis showed that relevant genes likely participated mTOR signaling pathway (Fig. 4D). Then, the correlation between LAMTOR2/mTOR and VSIG2 was qualitatively analyzed by endogenous co-immunoprecipitation, which indicated that VSIG2 can interact with LAMTOR2 and mTOR in PDAC cells (Fig. 4E, F and Fig. S1D, E). Besides, it was observed by immunofluorescence that VSIG2 respectively co-located with LAMTOR2 and mTOR in the cytoplasm of PDAC cells (Fig. 4G, Fig. S1F).

### VSIG2 acted as a scaffold to increase LAMTOR2 interaction with mTOR and enhance LAMTOR2-mediated mTOR activation

**Fig. 5:**
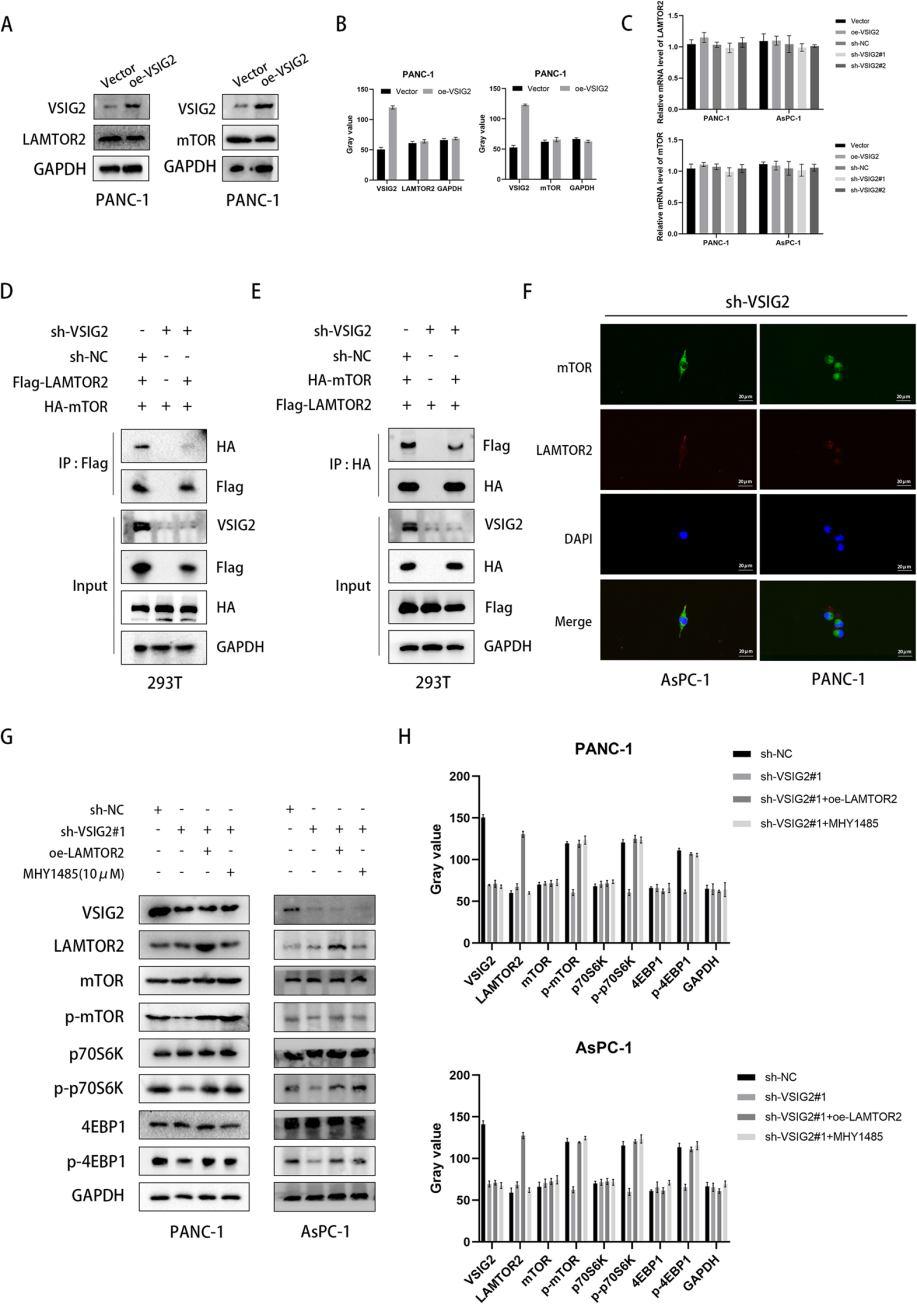
VSIG2 promoted the interaction between LAMTOR2 and mTOR, thereby increasing the activation of mTOR signaling pathway via LAMTOR2.** A**, **B** Overexpression of VSIG2 detected by immunoblotting in PANC-1 cells had no effect on protein expression of LAMTOR2 and mTOR. GAPDH was used as a loading control. **C** Knockdown and up-regulation of VSIG2 exerted no influence on mRNA expression of LAMTOR2 and mTOR, which were clarified by RT-qPCR in PANC-1 and AsPC-1 cells. **D**, **E**, **F** The level of interaction between LAMTOR2 and mTOR was testified through knockdown of VSIG2 and transfection of Flag-tagged as well as HA-tagged plasmids in 293T cells via further Co-IP assay and immunofluorescence. GAPDH was used as a loading control. **G**, **H** After VSIG2 knockdown, LAMTOR2 overexpression and mTOR agonist MHY1485 were added in PANC-1 and AsPC-1 cells, the expression and activation of mTOR and downstream key molecules were detected by western blots. GAPDH was used as a loading control

We observed that ectopic expression of VSIG2 had no significant effect on LAMTOR2 and mTOR (Fig. 5A-C and Fig. S1G). Then, it was recognized that VSIG2 depletion led to decreased co-immunoprecipitated proteins, which proved that VSIG2 affected the interaction between LAMTOR2 and mTOR (Fig. 5D-F). Through assessing the related indicators in mTOR signaling pathway, we found that VSIG2 inhibition suppressed the phosphorylation of mTOR and down stream molecules, while supplement of mTOR activator (MHY1485) or LAMTOR2 elevation recovered the phosphorylation of above molecules (Fig. 5G, H). Together, we observed that VSIG2 function as a scaffold to enhance the interaction between LAMTOR2 and mTOR, thereby inducing mTOR phosphorylation and activation.

### VSIG2 facilitated the progression of PDAC through LAMTOR2-mediated mTOR activation

**Fig. 6:**
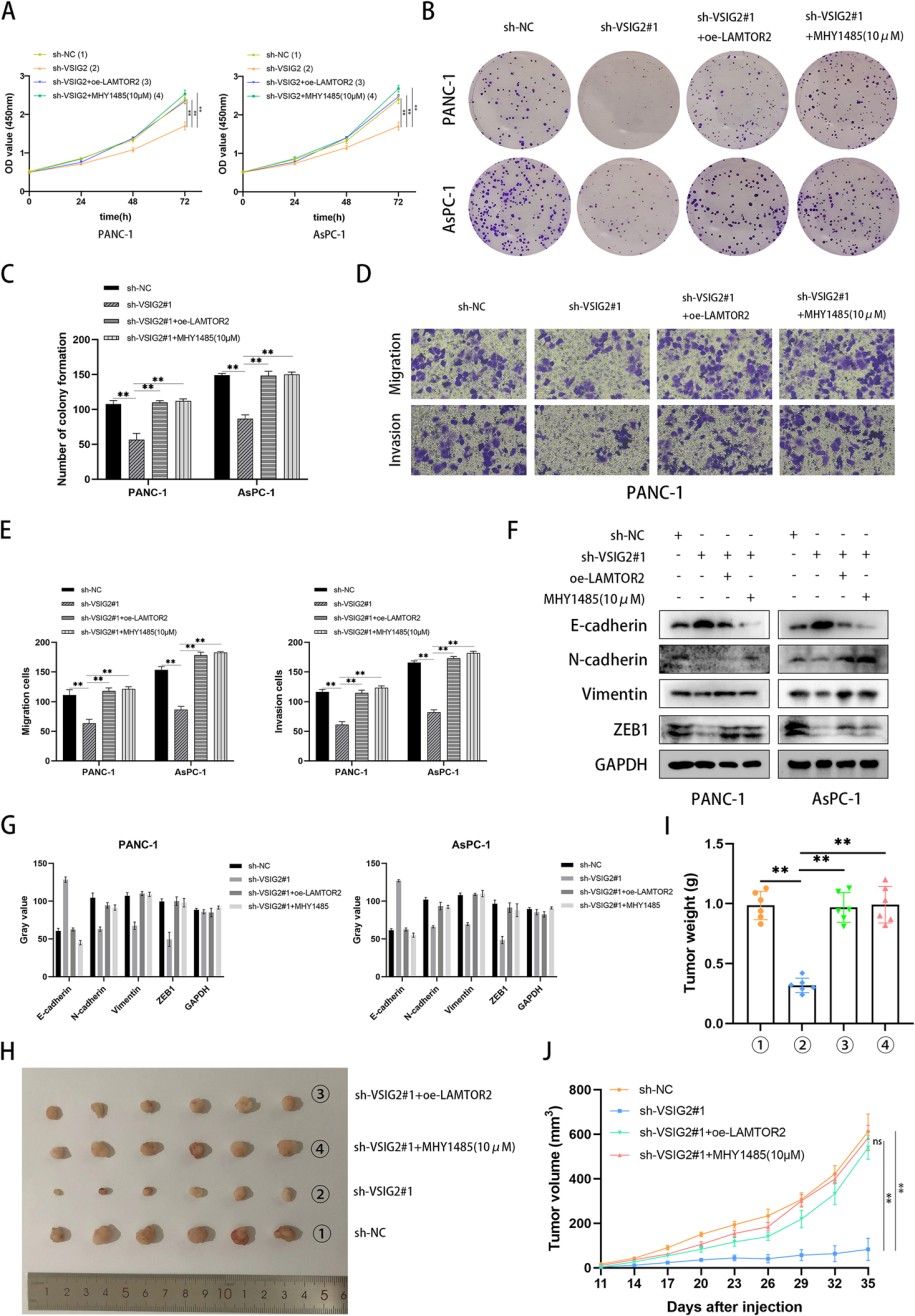
VSIG2 advanced PDAC progression through LAMTOR2-mediated mTOR activation. **A** CCK-8 assays were performed to detect the proliferation of PDAC cells regarding to down-regulation of VSIG2 and simultaneous elevation of LAMTOR2 or supplement of MHY1485, **P <0.01. **B**, **C** The properties of proliferation about PDAC cells according to different groups were demonstrated using colony formation assays, **P <0.01. **D**, **E** Transwell assays were used to certify the ability of migration and invasion about PANC-1 cells regarding to down-regulation of VSIG2 and simultaneous elevation of LAMTOR2 or supplement of MHY1485. **F**, **G** The related indicators of EMT were clarified by western blots after VSIG2 knockdown, LAMTOR2 overexpression and addition of mTOR activator MHY1485 in PANC-1 and AsPC-1 cells. GAPDH was utilized as a loading control. The bar charts showed statistical differences connected with migration and invasion PDAC cells, **P<0.01. **H**, **I**, **J** Subcutaneous xenograft tumor model was constructed to ulteriorly prove the conclusion, and tumor diameter was recorded once every three days, while tumor weight was investigated after dissection, **P<0.01

The result of CCK-8 showed that up-regulated LAMTOR2 or supplement of MHY1485 recovered repressed proliferation of PDAC cells induced by VSIG2 depletion (Fig. 6A). There were more colonies formed in LAMTOR2-overexpression cells or after addition with MHY1485, indicating that the property of proliferation about PDAC cells got enhanced (Fig. 6B, C). Furthermore, the results of Transwell and scratch assays showed that damaged invasion and migration abilities of PDAC cells resulted from VSIG2 knock-down were reversed by LAMTOR2 overexpression and supplement of MHY1485 (Fig. 6D, E and Fig. S1H). The epithelial indicator E-cadherin was decreased in LAMTOR2-overexpression cells, whereas mesenchymal indicators including N-cadherin, Vimentin, ZEB1 were elevated. Supplement of MHY1485 in PDAC cells displayed similar phenotypes, which recovered VSIG2 depletion-mediated EMT progress inhibition (Fig. 6F, G). The experimental conclusion about LAMTOR2-mediated VSIG2 oncogenic effect on PDAC was ulteriorly elucidated using subcutaneous xenograft tumor model (Fig. 6H). The record of tumor volume disclosed that knock-down of VSIG2 would inhibit the rate of tumor growth, and contribute to smaller volume at day 35. Nonetheless, over-expressed LAMTOR2 or supplement of MHY1485 accelerated tumor growth (Fig. 6J). Besides, tumor weight was recorded and analyzed after dissection, the role of VSIG2 depletion in tumor weight was impaired after elevation of LAMTOR2 or supplement of mTOR activator, indicating that VSIG2 advanced PDAC progression through LAMTOR2-mediated mTOR activation (Fig. 6I).

### Overexpression of VSIG2 in PDAC was associated with poor prognosis for PDAC patients

**Fig. 7:**
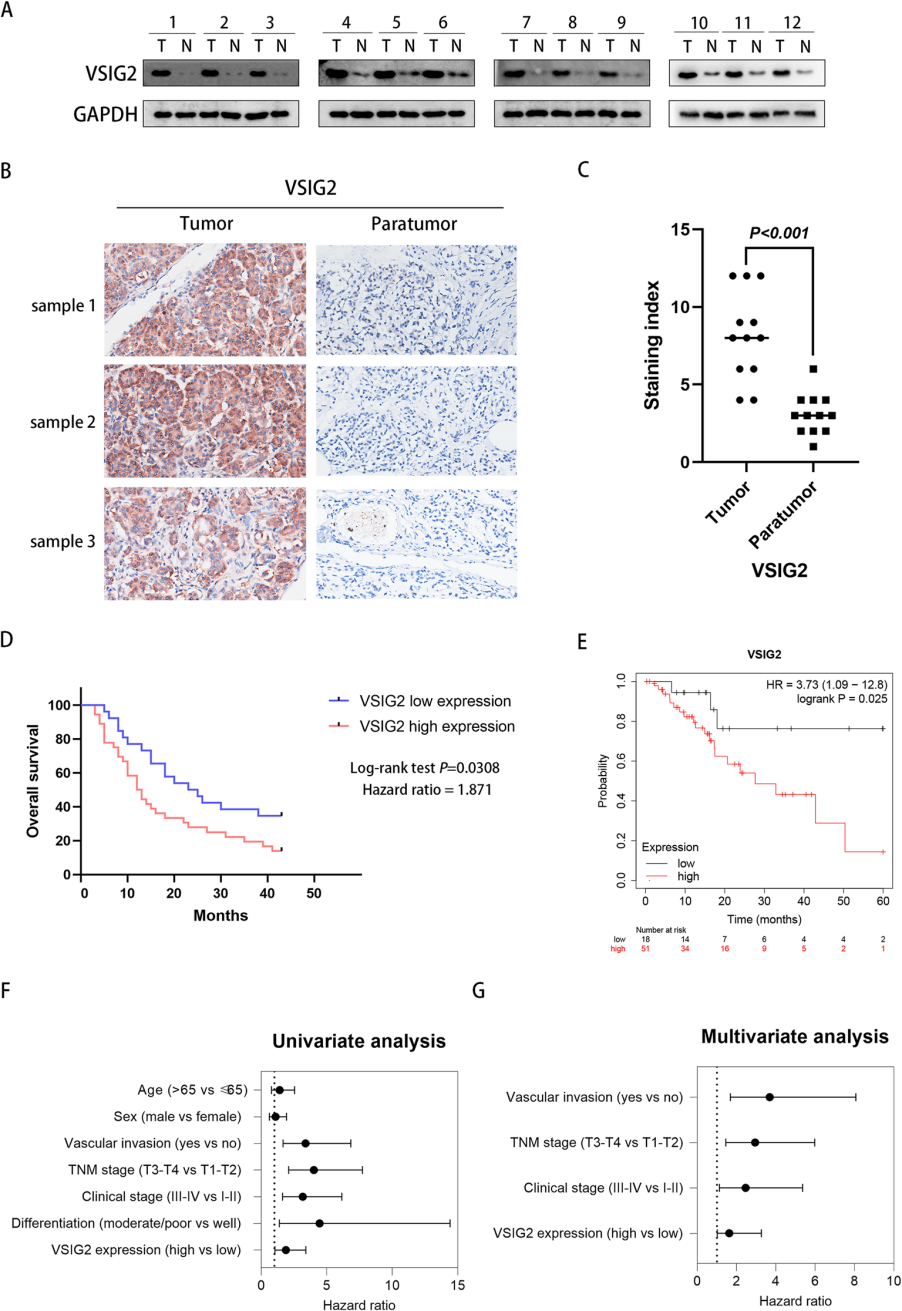
Overexpression of VSIG2 was associated with poor prognosis of PDAC patients. **A** Western blotting was performed on 12 pairs of pancreatic and para-cancerous tissues collected from PDAC patients, when GAPDH was served as a loading control. **B** 3 pairs of tumor and para-tumor tissues collected from PDAC patients were compared by immunohistochemistry (IHC) assay. **C** Scatter diagram of staining index about 12 pairs PDAC and adjacent tissue samples using 12-tier IHC scoring. **D** Overall survival curve was drawn through observing and recording the condition of PDAC patients for 43 months, while all PDAC patients were divided into two groups by VSIG2 expression level. **E** Disease-free survival probability of PDAC patients with different expression level of VSIG2 was acquired by Kaplan-Meier (https://kmplot.com/analysis/) database. **F** Forest plot of univariate Cox analysis regarding to multiple parameters. **G** Forest plot of multivariate Cox analysis regarding to multiple parameters

Twelve pairs of cancer and adjacent normal tissues were randomly selected from the samples of PDAC patients for Western blotting analysis, which showed that VSIG2 was overexpressed in tumor tissues, while the protein expression in adjacent tissues was extremely low (Fig. 7A). Subsequently, IHC was performed to further clarify VSIG2 expression in cancer and para-cancer tissues. PDAC tissues exhibited more intense immunostaining, instead that adjacent normal tissues showed less (Fig. 7B, C). Clinicopathological analysis according to the information of 62 patients with PDAC unfolded that VSIG2 overexpression was related to vascular invasion (*P*=0.024), and the expression level of VSIG2 was positively associated with tumor size (*P*<0.01). Additionally, PDAC patients with high VSIG2 expression were in advanced clinical stage with large probability (*P*<0.01). It was also observed that there was a poor cell differentiation when VSIG2 was highly expressed in PDAC cells (*P*=0.018). Nonetheless, there was no significant correlation between VSIG2 expression level and gender as well as age of PDAC patients (*P*=0.894 and 0.850, respectively) (Table 1). To evaluate the value of VSIG2 in reflecting the prognosis of patients with PDAC, we analyzed the relationship between VSIG2 expression level and the survival rate of 62 patients with PDAC in combination with bioinformatic prediction. The result of Kaplan-Meier analysis exhibited that survival rates were lower in patients with high VSIG2 expression than that in specimens with low expression, regardless of disease-free survival or overall survival (Fig. 7D, E). Further, Cox regression analysis showed that VSIG2 could serve as an independent risk factor which had impact on PDAC prognosis (Table 2 and Fig. 7F, G). Therefore, VSIG2 was expected to be the underlying prognosis biomarker for PDAC patients.

**Table 1:** Correlation between VSIG2 expression and clinicopathologic characteristics of PDAC

**Parameters**	**VSIG2 expression**		***n***	***χ***^***2***^	***P* value**
	**Low (*n*=26)**	**High (*n*=36)**			
Age (years)				0.036	0.850
≤65	10	13	23		
>65	16	23	39		
Sex				0.018	0.894
Male	14	20	34		
Female	12	16	28		
Vascular invasion				5.067	**0.024**
Yes	14	29	43		
No	12	7	19		
TNM stage				9.836	**<0.01**
T1-T2	16	8	24		
T3-T4	10	28	38		
Clinical stage				9.647	**<0.01**
I-II	15	7	22		
III-IV	11	29	40		
Differentiation				5.555	**0.018**
Well	7	2	9		
Moderate/Poor	19	34	53		

Bold values are statistically significant

*P*<0.05

**Table 2:** Univariate and multivariate Cox analysis of overall survival in PDAC patients

**Parameters**	**Univariate analysis**			**Multivariate analysis**		
	**HR**	**95% CI**	***P* value**	**HR**	**95% CI**	***P* value**
Age (>65 vs ≤65)	1.407	0.777-2.547	0.259	-	-	-
Sex (male vs female)	1.090	0.616-1.929	0.767	-	-	-
Vascular invasion (yes vs no)	3.376	1.665-6.846	**<0.001**	3.690	1.687-8.071	**0.001**
TNM stage (T3-T4 vs T1-T2)	4.021	2.090-7.736	**<0.001**	2.946	1.451-5.982	**0.003**
Clinical stage (III-IV vs I-II)	3.165	1.627-6.156	**<0.001**	2.461	1.130-5.359	**0.023**
Differentiation (moderate/poor vs well)	4.459	1.378-14.423	**0.013**	2.245	0.625-8.060	0.215
VSIG2 expression (high vs low)	1.882	1.039-3.411	**0.037**	1.632	1.018-3.259	**0.019**

*HR* hazard ratio, *CI* confidence interval

Bold values are statistically significant

*P*<0.05

## Discussion

Most patients with PDAC have been in the advanced stage of lack of surgical indications, and there is a poor effect of clinical treatment for PDAC due to its tolerance to traditional chemotherapy. Currently, the main clinical methods used in the diagnosis for PDAC include computed tomography (CT), magnetic resonance imaging (MRI), endoscopic ultrasonography (EUS), endoscopic retrograde cholangiopancreatography (ERCP) and magnetic resonance cholangiopancreatography (MRCP) [26, 27]. Given the disease detection rate is not ideal through above methods, it is still necessary to identify novel tumor markers with strong specificity and sensitivity, and rely on the detection methods including serum immunological examination and other laboratory tests to improve the early screening rate and diagnosis rate of PDAC. In addition to Carbohydrate antigen 19-9 (CA19-9), carcinoembryonic antigen (CEA) and CA12-5, which are the most clinically applicable biomarkers for PDAC [28, 29], transcriptomic, proteomic, and metabolomic biomarkers such as exosomes, miRNAs, proteins, and lipid metabolites have been screened by liquid biopsies from body fluids including blood, saliva, urine, and pancreatic juice in recent years [30, 31].

Insa M. Schmidt et al. found that VSIG2 expression was positively correlated to glomerular sclerosis and interstitial fibrosis/tubular atrophy (IFTA) when they analyzed the association between plasma protein biomarkers and histopathologic lesions in kidney [20]. Besides, Andreas Casselbrant et al. observed that VSIG2 function as protein biomarker which was significantly altered in patients with coronary artery disease (CAD) [32]. Further, VSIG2 was identified as a significantly dysregulated differential protein in colorectal cancer (CRC) through iTRAQ labeling proteomics and TMT labeling phospho-proteomics analysis of CRC tissues and adjacent normal tissues [33]. In our study, we elucidated that VSIG2 played the carcinogenesis role in PDAC as a differentially expressed oncogene through experimental verification combined with bioinformatics analysis. Specially, VSIG2 was uniquely up-regulated in PDAC, while VSIG2 had a low expression compared to non-cancerous tissues in multiple malignancies, which implied that VSIG2 could serve as a diagnostic biomarker and therapeutic target for PDAC.

VSIG2 is mainly composed of IgC2 and IgV domains, which belongs to IgSF and has structural similarity with B7 family proteins [14, 34]. Additionally, VSIG2 has been reported to exert immunosuppression effect as a type I transmembrane protein which is expressed in both immune and nonimmune cells [14]. In the occurrence and development of tumors, VSIG2 shows diversity and complexity both in function and mechanism. Cui Z et al. demonstrated that VSIG2 acted as a tumor suppressor in COAD, which conversely enhanced immune supervision rather than induced tumor immune evasion [17]. In our study, mass-spectrum analysis, co-immunoprecipitation, immunofluorescence staining, and immunoblotting were performed to reveal subcellular position of VSIG2 in PDAC cells and clarify that VSIG2 mechanically enhanced the interaction between LAMTOR2 and mTOR, thereby activating mTOR signaling pathway and accelerating PDAC malignant progression.

Dysregulation of the mTOR signaling pathway is involved in multiple human diseases including cancer [35], and vast reports have verified that the activation of mTOR participates the progression and chemoresistance of PDAC. mTORC1, which plays the vital and central role in mTOR signaling pathway comprises mTOR, Raptor and mLST8 protein. When cytoplasmic amino acid levels are sufficient, the Ragulator complex composed of LAMTOR1-5 is localized to the lysosome membrane by post-translational modification of LAMTOR1 [36], while the C-terminal L^154^VV^156^ motif of LAMTOR1 interacts with the C-terminal domain of Rag GTPase [36]. At this time, Ragulator activates Rag GTPase as a guanylate exchange factor [36]. The Ragulator-Rag complex acts as a scaffold to recruit mTORC1, thereby promoting Rheb-mediated mTORC1 activation [9, 36].

Structurally, LAMTOR1 wraps LAMTOR2-LAMTOR3 and LAMTOR4-LAMTOR5 roadblock protein pairs in a belt-like structure, and there is hydrophobic interaction between LAMTOR1 and other proteins [36]. LAMTOR2 and LAMTOR3 interact with each other through larger β-sheet and hydrophobic force, as do LAMTOR4 and LAMTOR5 [36]. Various secondary structures, including α-helices and β-sheets, are present in all component proteins. However, LAMTOR4 and LAMTOR5 lack α3 helices, so the α3 helices in LAMTOR2-LAMTOR3 subcomplex are momentous for stabilizing Ragulator complexes [36].

**Fig. 8:**
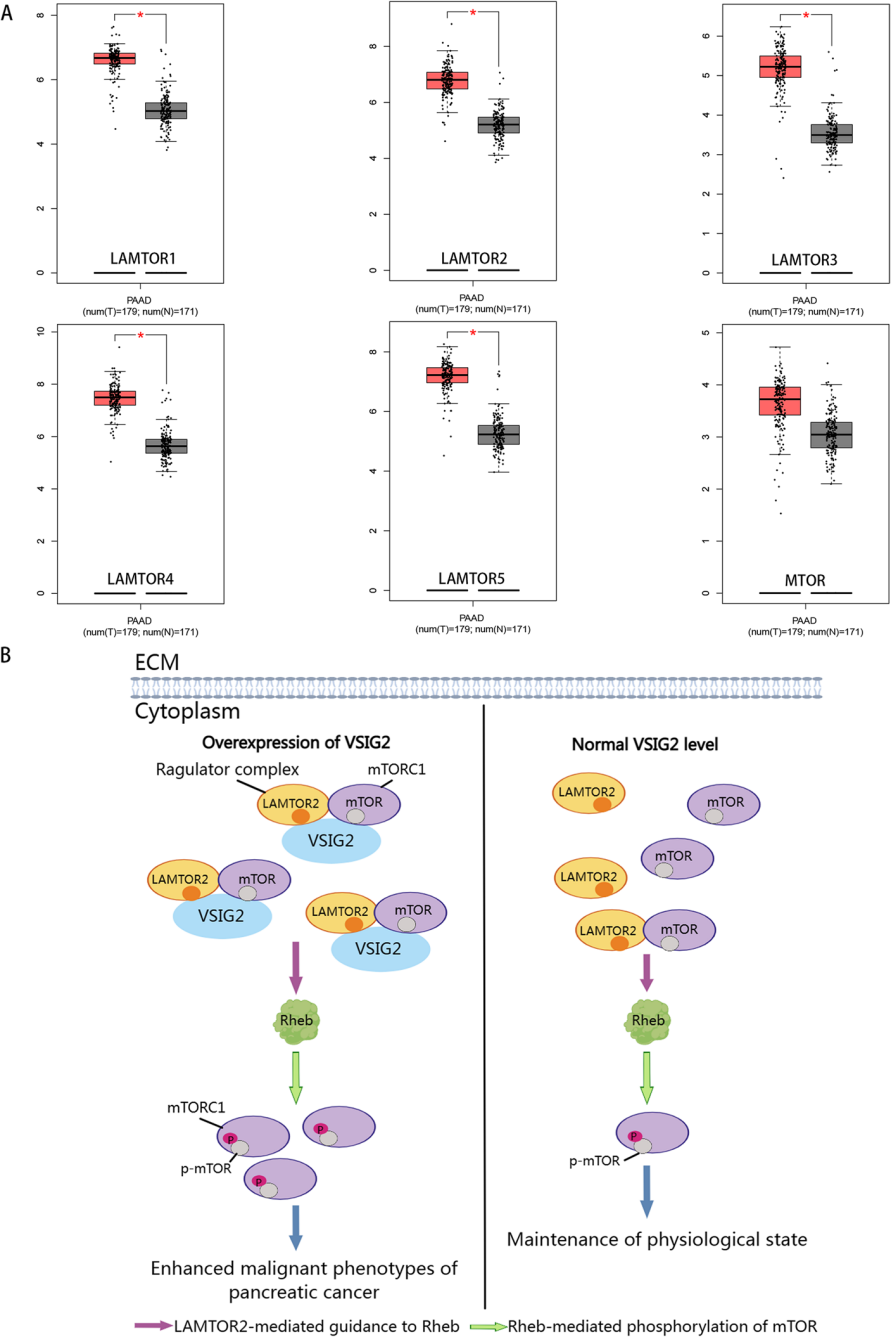
VSIG2 phosphorylated mTORC1, which contained mTOR, through the oncogenic effects of the Ragulator complex containing LAMTOR2. **A** GEPIA (http://gepia.cancer-pku.cn/) database predicted the component sections of Ragulator complex including LAMTOR1, LAMTOR2, LAMTOR3, LAMTOR4, LAMTOR5 were all highly expressed in PDAC compared to normal tissues, while the expression of MTOR had no difference between PDAC and normal pancreas tissues. **B** Compared with the expression level of VSIG2 under normal physiological conditions, when the expression of VSIG2 in PDAC cells was upregulated, VSIG2 can act as a scaffold to simultaneously play a recruitment effect on Ragulator and mTORC1, thus enhancing the interaction between mTORC1 and Ragulator complex including LAMTOR2. Subsequently, a large amount of mTORC1 was directed by Ragulator to the small GTPase Rheb, where phosphorylated activation of mTOR was increased and further initiated the malignant progression of PDAC

Through bioinformatics analysis, we found that the protein components of Ragulator complex, containing LAMTOR2, were significantly up-regulated in PDAC tissues, while there was no significant difference in the expression level of mTOR between PDAC tissues and normal pancreatic tissue (Fig. 8A). In view of the fact that Ragulator-mediated malignant biology of PDAC has not been reported so far, and according to the above functional properties of Ragulator, we speculated that Ragulator complex containing LAMTOR2 was dysregulated in PDAC cells, which can act as a cancer promoting factor to maintain the malignant phenotypes of PDAC by activating mTOR. In addition, combined with the rescue experiment in our study, the oncogenic mechanism of VSIG2 was likely to depend on the recruitment and activation function of Ragulator complex containing LAMTOR2, and VSIG2 can indirectly activate mTOR and downstream molecules through such mechanism, thus promoting the malignant progression of PDAC. In essence, VSIG2 enhanced the activation efficiency of mTOR in PDAC cells (Figs. 8B).

In conclusion, VSIG2, as a tumor-associated antigen, is expected to become a novel neoplasm marker in the immunological examination of PDAC, thereby improving the early screening rate and diagnosis rate of PDAC. Additionally, clinical correlation analysis confirmed that VSIG2 can be used as a prognostic marker for postoperative recurrence in patients with early PDAC. Further, the combination of mTOR inhibitors or the sole development of monoclonal antibodies and inhibitors regarding to VSIG2 can provide a new strategy for PDAC management by means of immunotherapy and gene therapy in the future.

### Supplementary Information

**Additional file 1: Fig. S1.** A Protein expression of VSIG2 in normal human pancreatic ductal epithelial (HPDE) and five PC cell lines including PANC-1, MIA PaCa-2, AsPC-1, SW 1990 and BxPC-3 were detected by western blotting. GAPDH was used as a loading control. B Transfection efficiency of VSIG2 knockdown in PANC-1 and AsPC-1 cells was clarified via western blotting, when GAPDH was served as a loading control. C Transfection efficiency of VSIG2 overexpression in PANC-1 and AsPC-1 cells was testified by immunoblotting, while GAPDH was performed as a loading control. D The interaction between VSIG2 and LAMTOR2 was testified by endogenic co-immunoprecipitation (Co-IP) in AsPC-1 cells. E The interaction between VSIG2 and mTOR was verified by endogenic Co-IP assay in AsPC-1 cells. F The interaction of VSIG2 with LAMTOR2 and mTOR and its spatial localization in AsPC-1 cells were observed by immunofluorescence. G Overexpression of VSIG2 detected by immunoblotting in AsPC-1 cells had no effect on protein expression of LAMTOR2 and mTOR. GAPDH was utilized as a loading control. H Transwell assays were utilized to testify the ability of migration and invasion about AsPC-1 cells regarding to down-regulation of VSIG2 and simultaneous elevation of LAMTOR2 or supplement of MHY1485.

**Additional file 2.**

**Additional file 3.**

## Data Availability

The data used to support the findings of this study are available from the corresponding author upon request.

## Abbreviations

FECD: Fuchs endothelial corneal dystrophy
IBS-D: Irritable bowel syndrome
AML: Acute myeloid leukemia
BRCA: Breast invasive carcinoma
ESCA: Esophageal carcinoma
HNSC: Head and neck squamous cell carcinoma
KIRC: Kidney renal clear cell carcinoma
KIRP: Kidney renal papillary cell carcinoma
LUAD: Lung adenocarcinoma
LUSC: Lung squamous cell carcinoma
PAAD: Pancreatic adenocarcinoma
STAD: Stomach adenocarcinoma
UCEC: Uterine corpus endometrial carcinoma
THCA: Thyroid carcinoma
READ: Rectum adenocarcinoma
PRAD: Prostate adenocarcinoma
LIHC: Liver hepatocellular carcinoma
GBM: Glioblastoma multiforme
COAD: Colon adenocarcinoma
