# Supplementary material for: VSIG2 promotes malignant progression of pancreatic ductal adenocarcinoma by enhancing LAMTOR2-mediated mTOR activation
Source: Cell Commun Signal. 2023 Aug 25;21:223. doi: 10.1186/s12964-023-01209-x (PMC10463957; doi:10.1186/s12964-023-01209-x)
Supplement: Supplementary file 2 — Additional file 1: Fig. S1. A Protein expression of VSIG2 in normal human pancreatic ductal epithelial (HPDE) and five PC cell lines including PANC-1, MIA PaCa-2, AsPC-1, SW 1990 and BxPC-3 were detected by western blotting. GAPDH was used as a loading control. B Transfection efficiency of VSIG2 knockdown in PANC-1 and AsPC-1 cells was clarified via western blotting, when GAPDH was served as a loading control. C Transfection efficiency of VSIG2 overexpression in PANC-1 and AsPC-1 cells was testified by immunoblotting, while GAPDH was performed as a loading control. D The interaction between VSIG2 and LAMTOR2 was testified by endogenic co-immunoprecipitation (Co-IP) in AsPC-1 cells. E The interaction between VSIG2 and mTOR was verified by endogenic Co-IP assay in AsPC-1 cells. F The interaction of VSIG2 with LAMTOR2 and mTOR and its spatial localization in AsPC-1 cells were observed by immunofluorescence. G Overexpression of VSIG2 detected by immunoblotting in AsPC-1 cells had no effect on protein expression of LAMTOR2 and mTOR. GAPDH was utilized as a loading control. H Transwell assays were utilized to testify the ability of migration and invasion about AsPC-1 cells regarding to down-regulation of VSIG2 and simultaneous elevation of LAMTOR2 or supplement of MHY1485. [file 12964_2023_1209_MOESM1_ESM.docx]

**Supplementary figure legends**

**
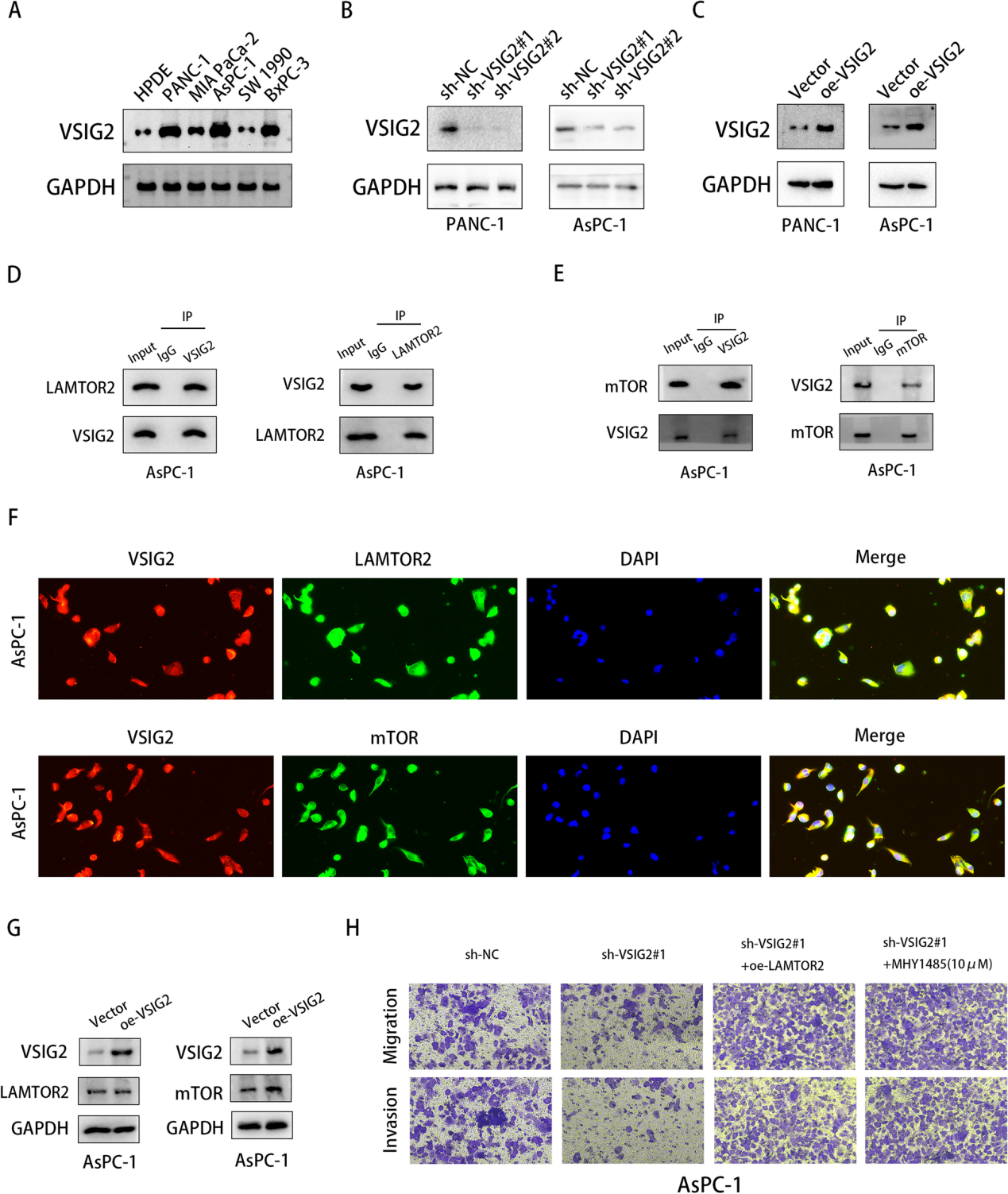
**

**Fig. S1 A** Protein expression of VSIG2 in normal human pancreatic ductal epithelial (HPDE) and five PC cell lines including PANC-1, MIA PaCa-2, AsPC-1, SW 1990 and BxPC-3 were detected by western blotting. GAPDH was used as a loading control. **B** Transfection efficiency of VSIG2 knockdown in PANC-1 and AsPC-1 cells was clarified via western blotting, when GAPDH was served as a loading control. **C** Transfection efficiency of VSIG2 overexpression in PANC-1 and AsPC-1 cells was testified by immunoblotting, while GAPDH was performed as a loading control. **D** The interaction between VSIG2 and LAMTOR2 was testified by endogenic co-immunoprecipitation (Co-IP) in AsPC-1 cells. **E** The interaction between VSIG2 and mTOR was verified by endogenic Co-IP assay in AsPC-1 cells. **F** The interaction of VSIG2 with LAMTOR2 and mTOR and its spatial localization in AsPC-1 cells were observed by immunofluorescence. **G** Overexpression of VSIG2 detected by immunoblotting in AsPC-1 cells had no effect on protein expression of LAMTOR2 and mTOR. GAPDH was utilized as a loading control. **H** Transwell assays were utilized to testify the ability of migration and invasion about AsPC-1 cells regarding to down-regulation of VSIG2 and simultaneous elevation of LAMTOR2 or supplement of MHY1485.
