# Supplementary material for: VSIG2 promotes malignant progression of pancreatic ductal adenocarcinoma by enhancing LAMTOR2-mediated mTOR activation
Source: Cell Commun Signal. 2023 Aug 25;21:223. doi: 10.1186/s12964-023-01209-x (PMC10463957; doi:10.1186/s12964-023-01209-x)
Supplement: Supplementary file 3 — Additional file 2. [file 12964_2023_1209_MOESM2_ESM.docx]

| Genes | Forward primer | Reverse primer |
| --- | --- | --- |
| VSIG2 | CATCTCTGAGTCCCATCCAATCC | TGACCCGCTTTGACTTAGAAC |
| LAMTOR2 | CTGCCATAGCCAGTAACATCTG | TAAACGCTTGGTTCCCGTTCC |
| MTOR | ATGCTTGGAACCGGACCTG | TCTTGACTCATCTCTCGGAGTT |
| β-actin | ACCAACTGGGACGACATGGAGAAAA | TACGGCCAGAGGCGTACAGGGATAG |
