# Supplementary material for: VSIG2 promotes malignant progression of pancreatic ductal adenocarcinoma by enhancing LAMTOR2-mediated mTOR activation
Source: Cell Commun Signal. 2023 Aug 25;21:223. doi: 10.1186/s12964-023-01209-x (PMC10463957; doi:10.1186/s12964-023-01209-x)
Supplement: Supplementary file 4 — Additional file 3. [file 12964_2023_1209_MOESM3_ESM.docx]

| Proteins | Catalogue |
| --- | --- |
| VSIG2 | PA5-86902, Thermo Fisher Scientific, USA |
| LAMTOR2 | PA5-103465, Thermo Fisher Scientific, USA |
| mTOR | 66888-1-Ig, Proteintech, Wuhan, China |
| GAPDH | 60004-1-Ig, Proteintech, Wuhan, China |
| Flag-tag | 66008-4-Ig, Proteintech, Wuhan, China |
| HA-tag | 66006-2-Ig, Proteintech, Wuhan, China |
| IgG | 30000-0-AP, Proteintech, Wuhan, China |
| E-cadherin | 60335-1-Ig, Proteintech, Wuhan, China |
| N-cadherin | 66219-1-Ig, Proteintech, Wuhan, China |
| Vimentin | 60330-1-Ig, Proteintech, Wuhan, China |
| ZEB1 | 66279-1-Ig, Proteintech, Wuhan, China |
| Phospho-mTOR | 67778-1-Ig, Proteintech, Wuhan, China |
| p70(S6K) | 66638-1-Ig, Proteintech, Wuhan, China |
| Phospho-p70(S6K) | 28988-1-AP, Proteintech, Wuhan, China |
| 4EBP1 | 60246-1-Ig, Proteintech, Wuhan, China |
| Phospho-4EBP1 | AP1334, ABclonal, Wuhan, China |
